# A Detection Algorithm for Citrus Huanglongbing Disease Based on an Improved YOLOv8n

**DOI:** 10.3390/s24144448

**Published:** 2024-07-10

**Authors:** Wu Xie, Feihong Feng, Huimin Zhang

**Affiliations:** 1 School of Computer Science and Information Security, Guilin University of Electronic Technology, Guilin 541004, China; wxie@guet.edu.cn (W.X.); guetffh@163.com (F.F.); 2 Guangxi Key Laboratory of Trusted Software, Guilin University of Electronic Technology, Guilin 541004, China; 3 Key Laboratory of Education Blockchain and Intelligent Technology, Ministry of Education, Guangxi Normal University, Guilin 541004, China; 4 Guangxi Key Lab of Multi-Source Information Mining and Security, Guangxi Normal University, Guilin 541004, China

**Keywords:** Citrus Huanglongbing, deep learning, object detection, YOLOv8n, orchard management

## Abstract

Given the severe impact of Citrus Huanglongbing on orchard production, accurate detection of the disease is crucial in orchard management. In the natural environments, due to factors such as varying light intensities, mutual occlusion of citrus leaves, the extremely small size of Huanglongbing leaves, and the high similarity between Huanglongbing and other citrus diseases, there remains an issue of low detection accuracy when using existing mainstream object detection models for the detection of citrus Huanglongbing. To address this issue, we propose YOLO-EAF (You Only Look Once–Efficient Asymptotic Fusion), an improved model based on YOLOv8n. Firstly, the Efficient Multi-Scale Attention Module with cross-spatial learning (EMA) is integrated into the backbone feature extraction network to enhance the feature extraction and integration capabilities of the model. Secondly, the adaptive spatial feature fusion (ASFF) module is used to enhance the feature fusion ability of different levels of the model so as to improve the generalization ability of the model. Finally, the focal and efficient intersection over union (Focal–EIOU) is utilized as the loss function, which accelerates the convergence process of the model and improves the regression precision and robustness of the model. In order to verify the performance of the YOLO-EAF method, we tested it on the self-built citrus Huanglongbing image dataset. The experimental results showed that YOLO-EAF achieved an 8.4% higher precision than YOLOv8n on the self-built dataset, reaching 82.7%. The F1-score increased by 3.33% to 77.83%, and the mAP (0.5) increased by 3.3% to 84.7%. Through experimental comparisons, the YOLO-EAF model proposed in this paper offers a new technical route for the monitoring and management of Huanglongbing in smart orange orchards.

## 1. Introduction

As one of the most important economic crops in the world [1], citrus trees are extremely vulnerable to diseases and pests during their growth. Citrus Huanglongbing is a type of citrus plant disease caused by Gram-negative, phloem-parasitic bacteria known as phloem bacilli. This disease often results in the gradual yellowing of citrus leaves, leaf margin curling, fruit deformity, and reducing the yield of citrus trees, which eventually leads to the death of citrus trees with huge losses to the citrus planting industry. It is also called citrus cancer [2]. Therefore, timely, rapid, and accurate diagnosis of trees infected with Huanglongbing is of great significance for the accurate prevention and control of citrus fruit tree diseases in order to reduce economic losses for diverse fruit farmers. Current detection methods of Huanglongbing usually include on-site diagnosis and laboratory biochemical analysis. On-site diagnosis requires manual observation of the appearance of the leaves and then relying on their accumulated experience to identify whether it is Huanglongbing. Although this is a simple and feasible method, it not only requires a lot of manpower and material resources but is also particularly prone to artificial misjudgment, which makes it difficult to meet the requirements for an accurate diagnosis of citrus Huanglongbing [3]. The use of biochemical analysis methods not only requires relevant technical personnel to master a vast amount of knowledge and skills but also relies on laboratory conditions and means of data collection. Furthermore, its scope of application is limited. At present, these two kinds of detection methods are either inefficient or costly, and it is difficult to effectively deal with the actual detection of citrus Huanglongbing on a certain scale, which is not suitable for the practical application scenarios of smart orange orchards. Fortunately, as one of the cores of information technology, the research and application of artificial intelligence technology are advancing by leaps and bounds. With the combination and development of artificial intelligence technology and agriculture, the research on the identification, detection, segmentation, and counting of crop diseases and insect pests in the field of agricultural precision planting has made some progress, which has advanced the development process of smart agriculture. Currently, the identification methods of applying artificial intelligence technology to the agricultural field are mainly divided into traditional image processing methods based on machine learning and novel processing methods that are based on deep learning [4].

Traditional image processing utilizes methods such as SIFT [5], SURF [6], and FAST [7] to solve simple problems. These methods do not require excessive code, perform the same operations on any image, require less computational resources, and thus possess strong generalizability. Compared with traditional image processing methods, deep learning neural networks are exploited to actively extract more abstract features for deep learning methods, which have stronger learning ability, more accurate prediction results, and can be used to handle more complex problems [8]. Consequently, deep learning methods are widely applied in the intelligent identification of crop pests and diseases in the field of precision planting in modern agriculture. The object detection networks based on deep learning methods are mainly divided into two types. One type is the two-stage object detection network based on region proposal, while the other type is the one-stage object detection network based on regression [9]. The two-stage object detection network generates a large number of candidate boxes in the first stage and then classifies and regresses the candidate boxes in the second stage. The representative network models are Faster-RCNN [10], Mask-RCNN [11], and HyperNet [12]. Different from the two-stage object detection network, the one-stage object detection network directly predicts the category and location of objects from the input images, which greatly improves the speed of inference, such as SSD [13], YOLO series [14,15,16], and other network models. Although the precision of the two-stage object detection network is relatively high, the two-stage object detection network requires many computing resources, and the running speed is relatively slow. On the contrary, the one-stage object detection network runs faster, but the precision still needs to be improved. Considering the real-time requirements of agricultural detection, the one-stage object detection network is more widely used in agriculture.

In recent years, the field of modern agricultural precision planting has attracted the interest of many researchers. Many methods of deep learning are utilized to accurately identify crop pests and diseases, which has also become an important research interdisciplinary direction. Based on deep learning, these researchers proposed various improved models, which further improved the detection accuracy and speed. For instance, Dou et al. [17] presented an improved CBAM-MobileNetV2 model to achieve real-time classification of citrus Huanglongbing disease severity. They applied the CBAM [18] attention module to capture semantic information of interest and the MobileNetV2 [19] module to reduce computation. In addition, they also fine-tuned the model parameters by means of transfer learning, which improved the accuracy of model classification and recognition. Lin et al. [20] came up with a refined NextViT [21] model, which was designed for orchard scenes such as fruit overlap or occlusion by dense leaves in citrus fruit detection. They used the GCFM (Global Context Fusion Module) to promote the interaction and fusion of local and global features so as to increase the detection ability of the model for occluded targets and improve the overall detection performance of the model. Lyu et al. [22] made improvements to the model based on YOLOv5s. They improved the modeling ability to focus on small targets by adding SE [23] attention to the backbone network and upgraded the CSP module in the neck network, thereby greatly enhancing the accuracy of the model for citrus psyllid detection. Jia et al. [24] proposed a deep learning method based on a convolutional neural network. They used AR-GAN data enhancement technology, transfer learning, and attention mechanisms to improve the classification ability of the model for citrus pests. On the basis of the Faster R-CNN model, Du et al. [25] proposed the pest R-CNN model to detect the fall armyworm pests in corn fields in the natural environment. They combined FPN [26], attention mechanism, deformable convolution, and multi-scale strategies. The experimental results indicated that the accuracy of the Pest R-CNN model had been greatly improved.

These neural networks based on deep learning can be used to achieve good recognition results to a certain extent and to provide a reference scheme for the detection of citrus Huanglongbing in this paper. However, during the detection process of citrus Huanglongbing, citrus Huanglongbing images often have a large number of healthy leaves, branches, and other occluded Huanglongbing leaves due to the characteristics of citrus trees growing in the natural environment. At the same time, there are many other diseases with similar colors. Leaves are covered by other objects, which is easy to cause problems such as clustering, overlapping, and occlusion, and object detection is difficult [27]. Therefore, the following aspects need to be considered for the disease identification process. First, the shapes and sizes of Huanglongbing leaves vary greatly. So, in the case of multiple targets of different scales in the Huanglongbing images, the neural network model finds it difficult to handle the information conflict between features of different scales. Secondly, Huanglongbing leaves often exhibit variations in brightness and color in images due to differences in their surface textures, as well as factors such as lighting and shadows, which subsequently impact the recognition results. Finally, the background in Huanglongbing recognition is often highly complex, with a large number of healthy leaves, other disease leaves of similar colors, branches, and other interfering information. This confusion of texture and color information in the background with the features of Huanglongbing leaves interferes with the accuracy of the recognition process.

For the reasons mentioned above, it is difficult to apply the target detection model to detect citrus Huanglongbing in the natural environment. Fortunately, YOLOv8 [28] is an outstanding model among one-stage object detection networks, which skillfully balances detection speed and accuracy, achieving efficient and precise object detection. YOLOv8 is an improved version derived from YOLOv5 [29]. Compared with YOLOv5, YOLOv8 employs the C2f module, which provides a richer gradient flow than the C3 module, further lightweighting the model while maintaining the CSP concept [30]. In addition, YOLOv8 adopts the Decoupled-Head [31] approach, which decouples the classification and regression tasks, making the training and inference of the network more efficient. This design helps improve the detection accuracy and speed of the model. Different from the anchor-based approach of YOLOv5, YOLOv8 abandons the anchor-based concept and adopts an anchor-free approach. This helps to simplify the model design and improve the detection accuracy. Meanwhile, in order to meet the demand for rapid detection in the field of smart agriculture, we specifically chose the smallest version of the YOLOv8 algorithm as our research object [32]. Therefore, this paper addresses this issue by taking YOLOv8n as the basic model and conducting specialized research and model optimization, considering the recognition scenario of citrus Huanglongbing under natural conditions. Aiming at the differences in surface texture of Huanglongbing leaves and the complex backgrounds in Huanglongbing images, we introduce an attention module into the backbone network of YOLOv8n in this paper in order to filter out interfering information and thereby enhance the model’s focus on the region of interest. At the same time, aiming to tackle the problem of the great variation in the shape and size of Huanglongbing leaves, we draw inspiration from the AFPN (asymptotic feature pyramid network) method [33] and adopt a similar structure to alleviate the problem of feature conflict from different levels, so as to retain useful information during the feature fusion processes. Based on the above improvements, we present a neural network model named YOLO-EAF that achieves higher detection accuracy through an appropriate attention module and an efficient multi-scale feature fusion structure. In this paper, the improvement of YOLOv8n and the innovative research work for the detection of Huanglongbing are summarized as follows:

(1) To improve the modeling ability to focus on citrus Huanglongbing, the EMA attention module is added to the backbone network of YOLO-EAF to enhance the model’s feature extraction ability.
(2) We introduce an adaptive spatial feature fusion module, which assigns different spatial weights to different levels of Huanglongbing features, enhances the importance of key levels, and reduces the impact of contradictory information from different scale objects.
(3) To accelerate the convergence of the model, improve the accuracy, and optimize the sample imbalance problem of the bounding box regression task, the Focal–EIOU is utilized to replace the existing loss function to improve the detection accuracy of the citrus Huanglongbing target.

## 2. Materials and Methods

### 2.1. Dataset

#### 2.1.1. Data Acquisition and Analysis

The citrus Huanglongbing image dataset used in this study consists of two parts: a small part of the data consists of 134 citrus Huanglongbing images collected from the Internet, while the remaining data were collected from the citrus orchard in Lingtian Town, Lingchuan County, Guilin, China. In this study, the data acquisition target was the leaves infected with citrus Huanglongbing disease. The images were captured using a handheld HUAWEI nova 7 Pro (Huawei Technologies Co., Ltd., Shenzhen, China), with an image resolution of 4608 pixels × 3456 pixels, and the images were saved in .jpg format. To ensure the diversity of the citrus Huanglongbing image dataset and make it closer to real detection scenarios, this dataset should include images of citrus leaves with Huanglongbing captured under different shooting distances, various lighting conditions, and different occlusion situations. Moreover, each image should contain multiple leaves infected with citrus Huanglongbing. Therefore, the data were photographed using a combination of different angles, distance, light intensity, and occlusion. After the collection work was completed, we took 744 images of citrus Huanglongbing disease. For training and evaluation, we divided the dataset into three parts with an 8:1:1 ratio: training set, validation set, and test set. The final dataset was divided into a training set containing 704 images, a validation set containing 87 images, and a test set containing 87 images.

#### 2.1.2. Image Annotation and Data Enhancement

**Figure 1:**
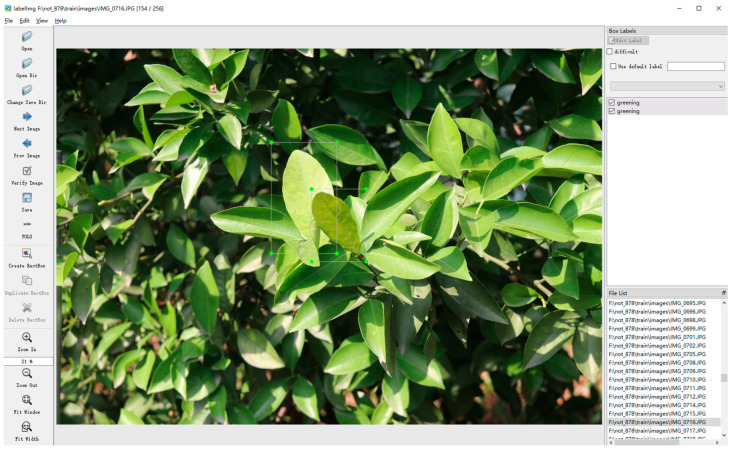
Labeled citrus Huanglongbing image.

The training of the citrus Huanglongbing image dataset is inseparable from the position data and category data of the Huanglongbing leaves in the image, which requires the annotation of the citrus Huanglongbing image. LabelImg 1.8.6 (as shown in Figure 1) software was used to label the leaves with Huanglongbing disease in the image one by one. The minimum external rectangular box was selected for labeling, and the .txt format file that can be recognized by the YOLO model was selected. The file includes the name of the corresponding image, the number of labels in the image, the index of the label category, the x and y coordinates of the center point of the rectangular box corresponding to the label, and the correspond to the values of w and h.

In the task of object detection, if the training image data are insufficient, it is easy to cause overfitting problems. Meanwhile, the citrus Huanglongbing images we collected are difficult to comprehensively reflect various conditions in natural environments, such as different light intensities, noise interference, image clarity, occlusion, and small objects, among other complex factors. To ensure the model performs well during training and testing, we adopted multiple data augmentation techniques to expand the citrus Huanglongbing images in the training and validation sets. These techniques include randomly adding Gaussian noise, adjusting brightness, cropping, translating, rotating, mirroring, and applying cutout. By applying these augmentation techniques to the images of citrus Huanglongbing, we can simulate the complex data distribution and noise in a natural orchard environment, thereby enhancing the generalization capability and practicality of the model [34]. The enhanced training set is expanded to 2816, the validation set is 348, and the test set is 87.

### 2.2. Related Theoretical Background

#### 2.2.1. The Principle of YOLOv8 Algorithm

**Figure 2:**
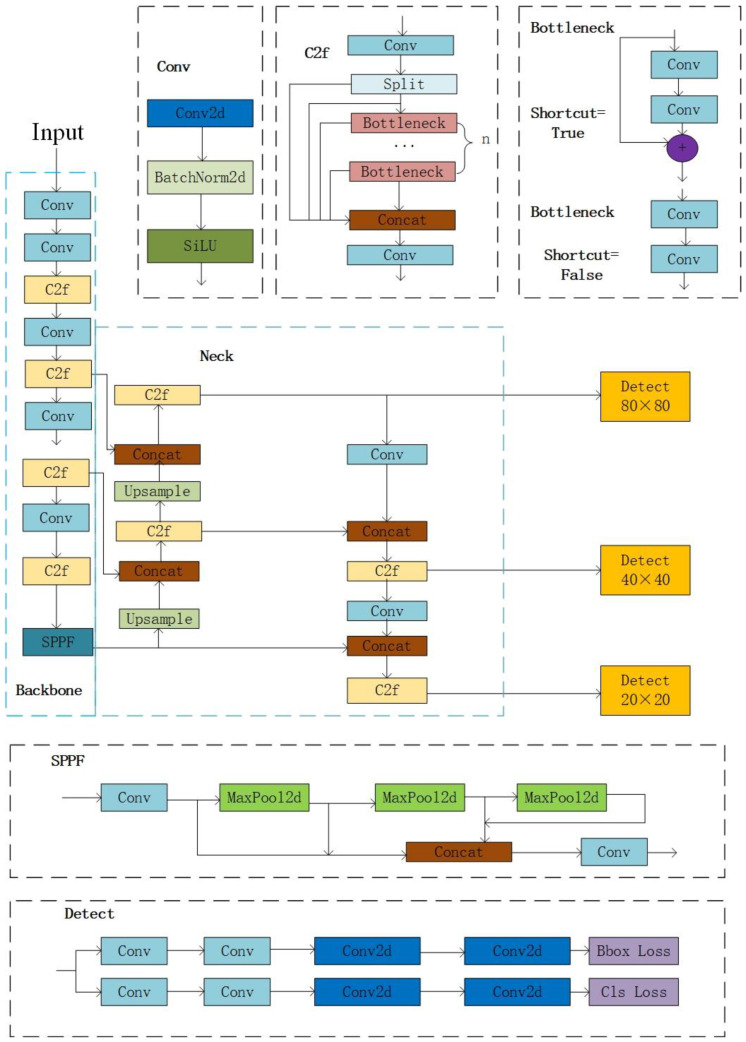
YOLOv8 network structure, the left part circled by the blue dashed line is the backbone, the right part circled by the solid blue line is the neck, and the parts circled by the gray dashed line are Conv, C2f, bottleneck, SPPF, and detect, respectively.

As a typical model of one-stage object detection, YOLO is widely used in real-time detection for its faster running speed. The YOLOv8 network is divided into YOLOv8n, YOLOv8s, YOLOv8m, YOLOv8l, and YOLOv8x according to the model depth multiple and layer channel multiple to meet the needs of different scenarios. YOLOv8 can be divided into four parts: input, backbone, neck, and head, and its structure is shown in Figure 2. The input is responsible for scaling the input image to the size required for training, including data enhancement operations such as scaling, changing image tone, and mosaic data enhancement. The backbone network is utilized to extract target features, which are composed of the convolution module Conv, C2f structure with richer gradient information, and the SPPF module used in YOLOv5. The neck is employed to enhance the fusion of features of different dimensions. Its structure is modeled after the feature pyramid networks (FPN) and path aggregation feature pyramid networks (PAFPN) architecture [35], with the exception of omitting the convolution operation in the up-sampling stage of YOLOv5. The head part is adopted to calculate the enhanced features and finally obtains the confidence and position of different objects.

#### 2.2.2. Attention Mechanism

**Figure 3:**
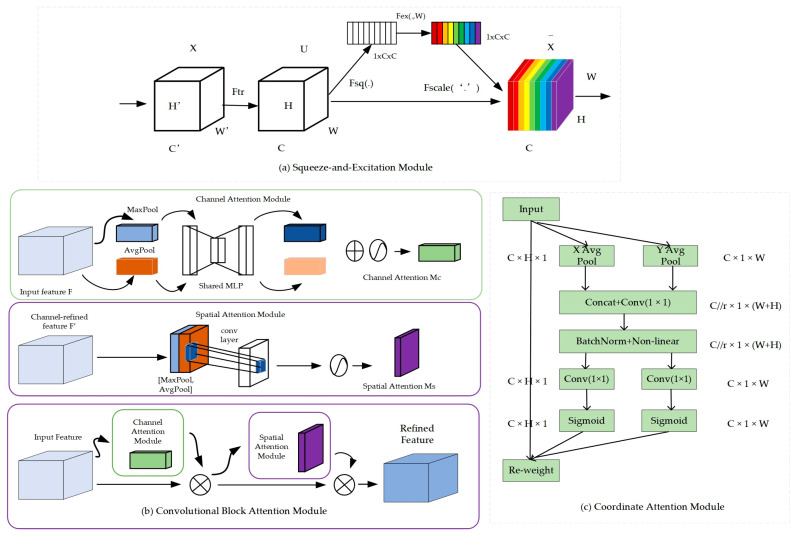
(**a**) The attention module structure of SE [23] mainly consists of two parts: Squeeze and Excitation. Different colors of the channels in the figure indicate the degree of contribution to the task, with darker colors indicating more importance. (**b**) CBAM [18] integrates channel and spatial attention to enhance network performance. (**c**) CA [36] utilizes positional information to optimize channel attention.

Attention mechanism is a key concept widely used in the field of deep learning, which is used to simulate the degree of attention to regions of interest in human visual and perceptual systems. At present, the attention mechanism methods are mostly based on the integration of channel or spatial information in different dimensions. Its essential role is to enhance the extraction of key features, so it is widely used in deep-learning neural networks to improve model accuracy. As a representative of channel attention, Squeeze-and-Excitation (SE, Figure 3a) networks employ an autonomous learning approach to capture the significance of each channel in the feature map. Subsequently, this significance is utilized to assign a weight to each channel, enabling the neural network to prioritize attention on the channels that are most beneficial for the current task. However, the SE attention mechanism only focuses on inter-channel information and ignores the importance of location in the coding process. The Convolutional Block Attention Module (CBAM, Figure 3b) is used to realize a sequential attention structure from channel to space, utilizing both the channel and spatial domains. It attends to the image in terms of both channels and space, thus enhancing the network’s feature representation ability. For CBAM, convolution is used to calculate spatial attention to establish spatial location information, but such convolution calculations can only be used to capture local relationships and cannot capture long-distance dependencies. Coordinate attention [36] (CA, Figure 3c) is generated through coordinate information embedding and coordinate attention. It not only enables CA to capture long-range dependencies within channels but also helps retain precise location information, allowing the network to localize targets more accurately. However, the importance of interactions is often ignored between spatial positions. The Efficient Multi-Scale Attention Module with cross-spatial learning (EMA) [37] further extends the CA attention mechanism by incorporating cross-spatial learning, which compensates for the shortcomings of the CA attention mechanism. The EMA mechanism is designed with versatility in mind, allowing it to be easily integrated into various existing neural network architectures, thereby enhancing the performance and efficiency of the models and making it applicable to a wide range of computer vision tasks.

#### 2.2.3. Feature Pyramid

**Figure 4:**
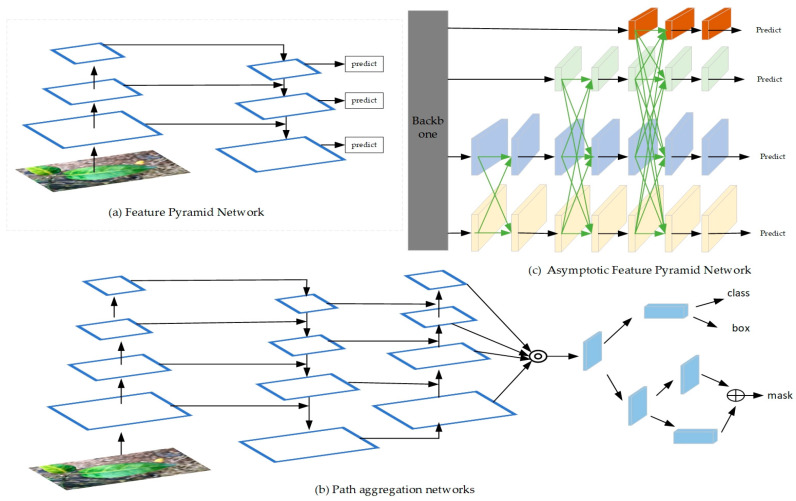
(**a**) FPN [26] processes features of different scales by constructing a feature pyramid. (**b**) PAFPN [35] strengthens the feature pyramid, improving multi-scale object detection and segmentation performance. (**c**) AFPN [33] enhances the accuracy and efficiency of object detection by strengthening interlayer interactions. Black arrows indicate convolution, and green arrows indicate ASFF.

In the field of computer vision, identifying objects of different sizes is a basic challenge. The feature pyramid above the image pyramid is the cornerstone of the standard solution [38]. Feature pyramid network (FPN, Figure 4a) fuses low-resolution, semantically strong features with high-resolution, semantically weak features through top-down paths and horizontal connections. This forms a feature pyramid that is semantically rich at all levels and can be constructed rapidly from a single input image scale. However, FPN directly fusing high-level features with low-level features can diminish the multi-scale representation capability. Path aggregation feature pyramid network (PAFPN, Figure 4b) enhances the connections and path aggregation of FPN by adding a bottom-up path, thus providing a more powerful multi-scale feature representation. However, the high-level or low-level features of PAFPN may be lost or degraded during propagation. Fortunately, the asymptotic feature pyramid network (AFPN, Figure 4c) is first used to fuse the low-level features from the backbone network and then gradually integrates the high-level features into the fusion process, which can avoid the large semantic gap between non-adjacent levels and retain useful information in the propagation process. Adaptive spatial feature fusion [39] (ASFF) plays a crucial role during the feature fusion process of AFPN. It avoids the contradictory information from non-adjacent hierarchical features by learning the relationship among different features.

### 2.3. The Architecture of YOLO-EAF

Although the original YOLOv8n model is powerful, it is still difficult for YOLOv8n to effectively detect citrus Huanglongbing targets with chaotic spatial distribution and complex background noise under natural environmental conditions, resulting in unsatisfactory detection performance. During the experimental process, we found that false detections mainly occurred in cases where the detection targets were small, heavily occluded, under excessive lighting conditions, or when Huanglongbing symptoms resembled other citrus diseases. Given these situations, we have conducted theoretical research and experimental analysis on YOLOv8n for the following three reasons. Firstly, during the process of continuous downsampling of citrus Huanglongbing images by the backbone in YOLOv8n, the interference information in the images becomes confused with the feature information of Huanglongbing, which affects the accuracy of recognition. Secondly, the neck part of YOLOv8n is utilized to enhance information propagation and preserve spatial information by adopting PAFPN. However, the high-level features of citrus Huanglongbing images from the backbone need to be propagated through multiple intermediate scales in the neck and interact with these intermediate features. During these processes, the semantics of high-level features may be lost or degraded. Finally, the loss function employed in the deep learning process of the current YOLOv8n is the CIOU [40] (Complete Intersection over Union) function. The CIOU function cannot truly reflect the true difference in the aspect ratio of the citrus Huanglongbing image and needs to be improved.

**Figure 5:**
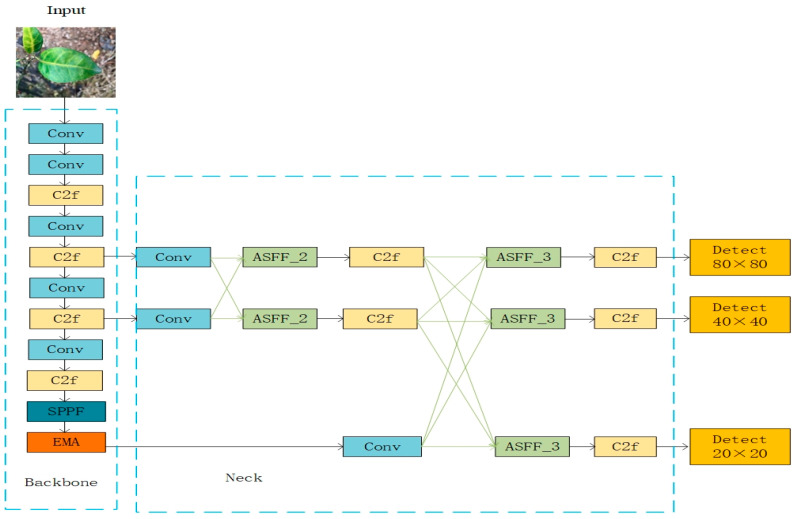
YOLO-EAF structure, the backbone and neck are circled by blue dashed frames. The Light blue represents Conv, light yellow represents C2f, dark blue represents SPPF, orange part is the EMA module, the light green part is the ASFF module, and light orange represents Detect.

Therefore, the improvement motivation of this method is to enhance the ability of the backbone of YOLOv8n to represent the target area, strengthen the retention of detailed information in the process of high-level or low-level feature propagation and interaction in the neck part, and improve the ability of the loss function to locate the target of citrus Huanglongbing. In order to adapt to the identification and detection of citrus Huanglongbing under natural and complex environments, the model can be used to learn local and global information and improve the accuracy of identifying citrus Huanglongbing. Based on the YOLOv8n model, we propose an improved model, namely the YOLO-EAF model, whose structure is shown in Figure 5. In order to enhance the capability of the backbone network to extract features and locate the target area more effectively, we added the EMA attention module under the SPPF module of the backbone. Then, to efficiently integrate features from different levels and avoid large semantic gaps between non-adjacent layer features, we adopted the ASFF module in the neck network. We also drew inspiration from the AFPN network structure, reducing the feature fusion of four layers in AFPN (as shown in Figure 4c, where black arrows represent convolutions and green arrows represent ASFF) to three layers and replacing all Conv modules after feature fusion through the ASFF module with C2f modules that have richer gradient flow. In addition, in order to accelerate the convergence of the model and improve the accuracy of the model, the original CIOU loss function was replaced by the Focal–EIOU [41] loss function.

From Figure 5, we can see that the overall process of YOLO-EAF is as follows. Firstly, the input citrus Huanglongbing image is extracted at the backbone to obtain three different scales of Huanglongbing feature maps. Secondly, the ASFF module in the neck network is employed to fuse the low-level features related to Huanglongbing first and subsequently fuse the high-level features related to Huanglongbing. This approach enhances the useful information at the key level and mitigates the issue of conflicting Huanglongbing information. Finally, the three Huanglong disease feature maps after feature fusion are used to predict the position and category in Detect. During the training processes, Focal–EIOU is used to locate the Huanglongbing target more accurately. Next, we describe the three improved modules in detail in Section 2.4.

### 2.4. Algorithm Improvement Methods

#### 2.4.1. EMA Attention Module

**Figure 6:**
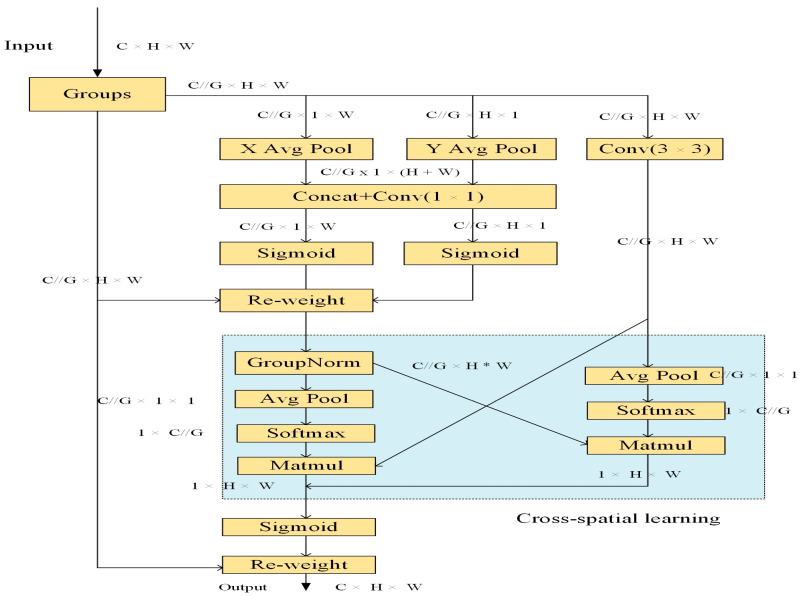
The overall structure of the EMA [37].

In order to enhance the feature extraction ability of the network for citrus Huanglongbing to more effectively locate specific targets in citrus Huanglongbing images, we tried to add an attention mechanism to YOLO-EAF. The EMA attention module has attracted great interest due to its novel features. EMA can not only be used to help the detection model identify the target of interest more accurately but also to eliminate the interference information from the images. Specifically, for EMA, a general method is adopted to reshape the channels into the batch dimension without using general convolution to achieve a certain form of dimensionality reduction, thus avoiding the loss of some detailed information. Additionally, EMA is used to deal with parallel sub-networks with two 1 × 1 branches to build local cross-channel interactions without performing channel dimensionality reduction, embedding precise positional information into the channels, and capturing long-range dependencies on the channels. In addition, EMA is also used to fuse the output feature maps of the two parallel sub-networks through cross-space learning methods to achieve richer feature aggregation. The structure flow of the EMA is shown in Figure 6.

It can be seen from Figure 6 that EMA mainly has three stages. The first is the feature grouping stage, for any given input features $X\in R^{C\times H\times W}$. EMA is utilized to divide X into *G* sub-features along the cross-channel dimension in order to learn distinct semantics, and the overall grouping is represented by Formula (1).

(1) $$X=\left[X_{0},X_{i},\ldots ,X_{G-1}\right],X_{i}\in R^{C/∕G\times H\times W}$$

The second is the parallel subnet stage. EMA is used to extract the attention weight of the grouping feature map through three parallel paths. The two parallel paths are on the 1 × 1 branch, and the third path is on the 3 × 3 branch. In the 1 × 1 branch, two one-dimensional global average pooling operations are used to encode the channels along two spatial directions respectively, so as to generate the attention weight maps along the horizontal and vertical directions, and then the two attention weight maps are multiplied with the input $X_{i}\in R^{C/∕G\times H\times W}$ to generate the enhanced new feature map. Only one 3 × 3 kernel is stacked in the 3 × 3 branch to capture multi-scale feature representations for later cross-space learning. In this way, the EMA is used not only to encode the information between channels to adjust the importance of different channels but also to retain accurate spatial structure information in the channels.

The third is the cross-spatial learning stage. The outputs from the 1 × 1 and 3 × 3 branches are globally average pooled to encode spatial information. After reshaping to corresponding dimensions, a matrix dot product operation is performed to obtain two spatial attention maps. Finally, these maps are fused to establish a dependency relationship between long and short distances.

#### 2.4.2. ASFF Adaptive Spatial Feature Fusion Module

**Figure 7:**
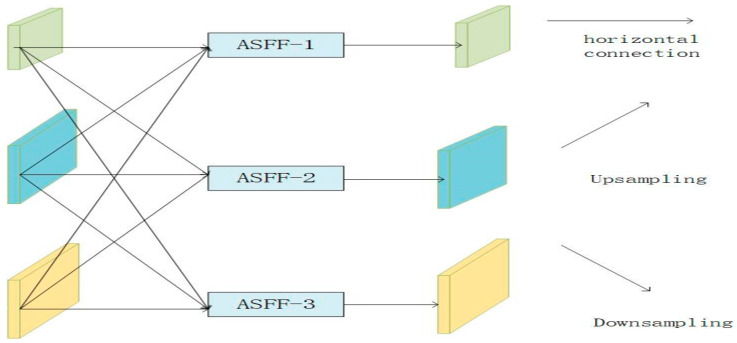
The three-level structure representation of ASFF [39]. The modules in light blue, light green, and light yellow in the figure are all convolutional modules.

During the processes of citrus Huanglongbing object detection, multi-scale features are very important for encoding targets with scale differences. Fortunately, adaptive spatial feature fusion (ASFF) can be used to assign different spatial weights to different levels of features in the multi-level feature fusion process, enhance the importance of key levels, and reduce the impact of contradictory information from different objects so that the fused feature network has higher recognition efficiency and accuracy. Therefore, in order to improve the fusion ability of multi-scale citrus Huanglongbing feature maps, they are obtained via the YOLO-EAF backbone network feature extraction. We introduced the ASFF module into the neck network of the YOLO-EAF model during Huanglongbing image object detection. The structure of the ASFF module used in this paper is shown in Figure 7.

In Figure 7, the ASFF module integrates three levels of features. $X_{ij}^{n\to l}$ represents the feature vector from the l layer to the n layer at the position (i,j), and the obtained feature vector is denoted as $y_{ij}^{l}$. The weighted feature fusion formula of ASFF is:

(2) $$y_{ij}^{l}=\alpha_{ij}^{l}*x_{ij}^{1\to l}+\beta_{ij}^{l}*x_{ij}^{2\to l}+\gamma_{jj}^{l}*x_{ij}^{3\to l}$$

(3) $$\alpha_{ij}^{l}+\beta_{ij}^{l}+\gamma_{jj}^{l}=1$$

In the formula, $\alpha_{ij}^{l}$, $\beta_{ij}^{l}$, $\gamma_{ij}^{l}$ denote the three spatial feature weights of the l layer, and the three intermediate feature weights are constrained by the Formula (3). During the process of object detection of citrus Huanglongbing, ASFF plays the role of feature fusion as follows. Firstly, ASFF utilizes 1 × 1 convolution and bilinear interpolation for upsampling and employs different convolutional kernels and strides for downsampling in order to adjust Huanglongbing disease features from different levels to the same scale. Secondly, in terms of spatial position, the feature of Huanglongbing carrying contradictory information should be filtered, and the corresponding spatial weights should be reduced. On the contrary, the feature of Huanglongbing carrying more distinguishing cues should be strengthened, and the corresponding spatial weights should be increased. Finally, different levels of Huanglong disease features are adaptively fused together in a feature-weighted manner. Therefore, ASFF can be used to solve the inconsistency problem between different scale features by learning the relationship between different Huanglong disease features so as to retain useful information for combination and improve the accuracy of network recognition of Huanglong disease targets for different scales.

#### 2.4.3. Focal–EIOU Loss Function

Under the natural environment, one of the important tasks of citrus Huanglongbing object detection is to determine the position of the Huanglongbing image boundary frame. Therefore, in order to improve the recognition accuracy of the model and accelerate its convergence speed, we improved the loss function of model training. For the original YOLOv8n, CIOU is used as the coordinate loss function. CIOU is an extension of DIOU (Distance Intersection over Union) loss that takes into account the aspect ratio of the predicted bounding box, which further improves the accuracy of the model. The calculation formula of CIOU is as follows:

(4) $${LOSS}_{CIOU}=1-IOU+\frac{\rho^{2}(b,b^{gt})}{C^{2}}+\alpha v$$

(5) $$\alpha =\frac{v}{\left(1-IOU\right)+v}$$

(6) $$v=\frac{4}{\pi^{2}}{\left(arctan\frac{w^{gt}}{h^{gt}}-arctan\frac{w}{h}\right)}^{2}$$

**Figure 8:**
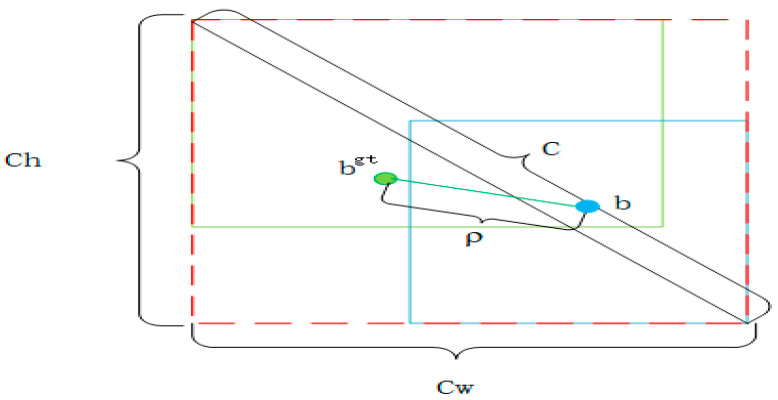
CIOU [40] computing structure representation diagram.

Among them, IOU is the intersection and union ratio of the prediction box and the real box. ρ represents the Euclidean distance between the two center points. b and $b^{gt}$ represent the center points of the prediction box and the real box, respectively, c represents the diagonal distance of the minimum closure area that can contain both the prediction box and the real box, α is suitable for the parameter to weigh, v is a parameter used to measure the consistency of the aspect ratio, and its structure is shown as Figure 8. However, CIOU reflects the difference in aspect ratio through v, rather than the true difference in confidence, so it sometimes hinders the optimization of similarity.

Therefore, in view of the problems existing in CIOU, Focal–EIOU calculates the difference in width and height on the basis of CIOU and introduces Focal Loss to focus on the high-quality anchor box. The expression of Focal–EIOU is as follows:

(7) $${LOSS}_{EIOU}=1-IOU+\frac{\rho^{2}\left(b,b^{gt}\right)}{C^{2}}+\frac{\rho^{2}\left(w,w^{gt}\right)}{C_{w}^{2}}+\frac{\rho^{2}(h,h^{gt})}{C_{h}^{2}}$$

(8) $${LOSS}_{Focal-Loss}={IOU}^{\gamma }+{Loss}_{EIOU}$$

In the formula, $C_{w}^{2}$ and $C_{h}^{2}$ are the squares of the width and height in the minimum bounding rectangle, and γ is the parameter to control the consistency of outliers. The EIOU loss function divides the loss term of the height–width ratio into the ratio of the predicted height–width to the minimum height–width of the outer frame, which accelerates the convergence speed of the model and improves the regression accuracy of the model. On the basis of EIOU, the Focal Loss constraint is introduced to optimize the sample imbalance problem in the bounding box regression task. That is, the optimization contribution of the anchor frame with low overlap with the target bounding box of citrus Huanglongbing to the bounding box regression is reduced so that the regression process is more focused on high-quality anchor frames.

## 3. Results and Analysis

### 3.1. Experimental Environment and Parameters

These experiments are based on the PyTorch 1.8.1 deep learning framework for training and testing. The hardware configuration is Intel (R, Intel Corporation, Santa Clara, CA, USA) Core (TM) i7-9700KCPU @ 3.60 GHz processor, 8 gb NVIDIA Quadro P4000 GPU. The operating system is Ubuntu 18.04.1, python 3.7, CUDA 11.4, cuDNN 7.6.5. During the whole training process, the image input size was 640 × 640. The Batch size was 16. The workers were set to 8. The initial learning rate was 0.01. The weight attenuation rate was 0.0005. The momentum factor was 0.937. The optimizer was SGD. The network training round was 300. The mosaic is closed in the last 10 rounds of training.

### 3.2. Model Evaluation Indicators

In order to evaluate the detection results of citrus Huanglongbing, in this paper, we use precision (P), recall (R), $F_{1}$-score ($F_{1}$), average precision (AP) and mean average precision (mAP), params (the number of trainable parameters in the model), Giga Floating Point Operations Per Second (GflOPs, representing the number of floating-point operations a computer can perform per second), and frames per second (FPS, representing the inference speed of the model) are among the metrics used for model evaluation.

(9) $$P=\frac{T_{p}}{T_{p}+F_{p}}\times 100\%$$

(10) $$R=\frac{T_{p}}{T_{p}+F_{N}}\times 100\%$$

(11) $$F_{1}=\frac{2PR}{P+R}\times 100\%$$

(12) $$AP=\int_{0}^{1}P(R)dR$$

(13) $$mAP=\frac{\sum_{i=1}^{n}AP_{i}}{n}$$

In the formula, $T_{p}$ denotes the number of positive samples that are predicted to be positive. $F_{p}$ represents the number of negative samples predicted to be positive. $F_{N}$ represents the number of positive samples that are predicted to be negative. $F_{1}$ is the harmonic average of precision and recall rate, which avoids the single extreme value of precision or recall rate and is used to comprehensively reflect the overall index. AP is the area under the PR curve. The mAP is the AP average value of citrus Huanglongbing, which represents the performance of the model’s overall detection accuracy for the disease and is of great significance for the evaluation of the object detection model.

### 3.3. Performance Comparison of Various Attention Mechanisms on YOLOv8n

**Figure 9:**
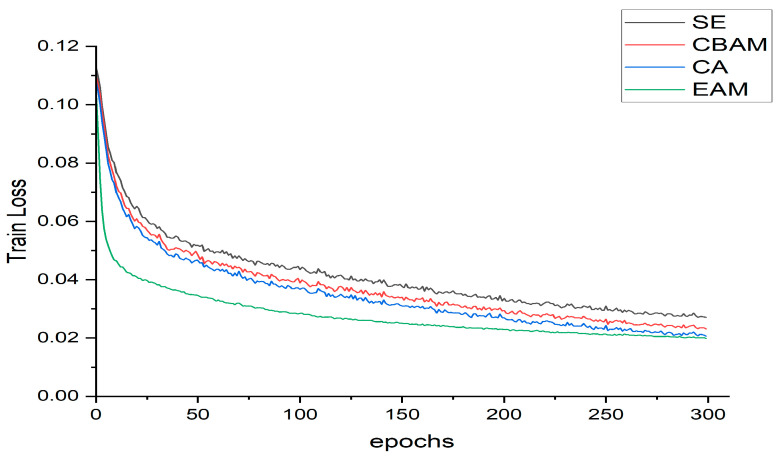
Loss curve of different attention modules.

Based on the YOLOv8n model, we integrated the EMA attention module and conducted an in-depth analysis of its performance through comparative experiments with SE, CBAM, and CA attention modules. The results are shown in Table 1 and Figure 9.

As can be seen from Table 1, under the premise of unchanged params and GFLOPs, the precision of EMA increased by 5, 6.1, and 9.5 percentage points compared to SE, CBAM, and CA attention modules, respectively. The mAP increased by 6.7, 5.5, and 4.2 percentage points, respectively, and the FPS increased by 3.48, 2.84, and 3.55 frames per second, respectively. Even though the recall rate of EMA decreased by 2 percentage points compared to the CA attention module, the overall performance of EMA is better than that of other attention modules. Furthermore, as shown in Figure 9, the loss curve of EMA drops rapidly and converges, showing a better trend than the loss curves of other attention modules. Therefore, in the YOLOv8n model, the EMA attention module demonstrates higher efficiency in filtering effective information and has significantly stronger recognition capabilities for Huanglongbing disease compared to the SE, CBAM, and CA attention modules.

### 3.4. Performance Comparison of Various Loss Functions on The YOLOv8n Model

To explore the impact of different loss functions on model performance, we adjusted the original YOLOv8n model by replacing its original CIoU loss function with MPDIOU, EIOU, and Focal–EIOU, respectively. Table 2 shows the application effects of these four loss functions on the YOLOv8n model.

**Figure 10:**
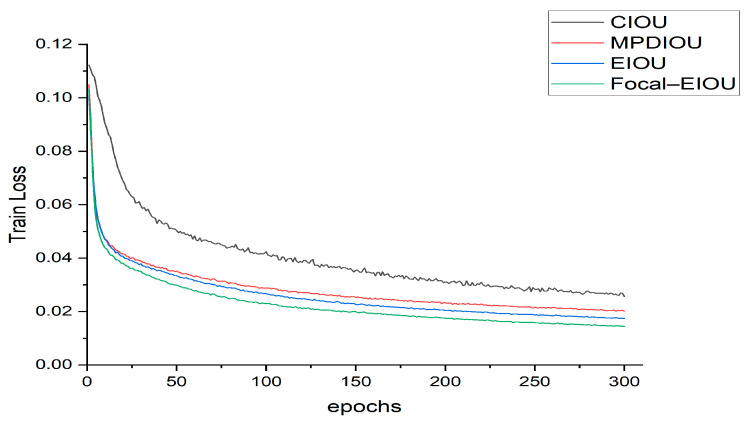
Loss curves of different loss functions.

The data show that when Focal–EIOU is used as the loss function, the overall accuracy of the model is 5.1, 4.2, and 0.2 percentage points higher than that of using CIOU, MPDIOU [42], and EIOU, respectively. The mAP(0.5) has also increased by 0.7, 0.5, and 0.2 percentage points, respectively, and the $F_{1}$-score has increased by 0.96, 0.66, and 1.54 percentage points, respectively. However, the number of model parameters (params) and computational complexity (GFLOPs) remain unchanged. In addition, Figure 10 depicts the loss curves of the four loss functions during model training. By observing these curves, we found that whether it is CIoU, MPDIOU, EIOU, or Focal–EIOU, they can all quickly reduce model loss and stably maintain it at a low level. However, Focal–EIOU’s loss decreases faster and has a smaller convergence loss. Overall, the comprehensive performance of using Focal–EIOU as the loss function is relatively obvious, and the detection accuracy is the highest.

### 3.5. Ablation Experiment

In order to further verify the performance of the improved algorithm proposed in this experiment, the same dataset is used for training and testing, and various improved methods are used for ablation experiments. Based on YOLOv8n, in this study, we propose a more effective network model: adding an EMA attention module, introducing an ASFF feature fusion structure, replacing the loss function, and conducting eight groups of experiments, respectively. The ablation test results are shown in Table 3. 

From Table 3, it can be seen that after adding the EMA attention module to the backbone, the mAP (0.5) of the detection model increased by 1 percentage point, indicating that after adding the EMA module, the ability to extract effective features of citrus Huanglongbing was enhanced. After introducing the ASFF feature fusion module in the neck part, mAP (0.5) is increased by 1.9 percentage points, which proves that ASFF can fuse the original multi-scale features extracted by more backbone networks and make the feature fusion more reasonable. After changing the CIOU loss function of YOLOv8n to Focal–EIOU, mAP (0.5) increased by 0.7 percentage points, indicating that replacing Focal–EIOU can be utilized to solve the conflict between the classification problem and the regression task of citrus Huanglongbing feature, which not only accelerates the convergence speed of the model but also improves the detection accuracy of the model. In general, the YOLO-EAF model proposed in this paper has an accuracy rate of 82.7%, which is 8.4 percentage points higher than the original YOLOv8n model. Although the recall rate decreased by 1.2 points, the mAP (0.5) increased by 3.3 percentage points. The above data show that compared with YOLOv8n, YOLO-EAF has stronger feature extraction and multi-scale feature fusion capabilities and has better performance in the detection of citrus Huanglongbing.

**Figure 11:**
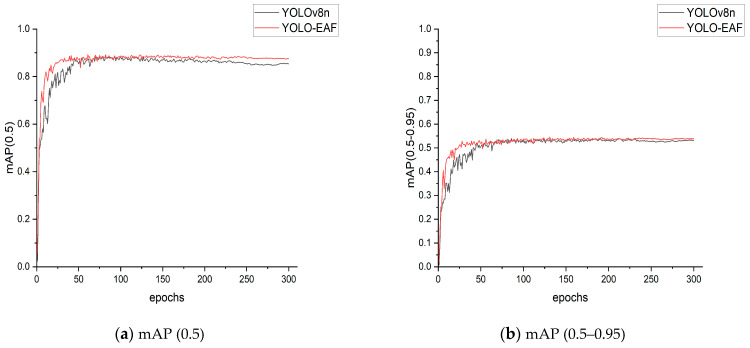
Comparison of mAP (0.5) and mAP (0.5–0.95) curves of the model before and after improvement.

Figure 11 shows the comparison curves of mAP (0.5) and mAP (0.5–0.95) on the training set and validation set of YOLOv8n and YOLO-EAF models. It can be seen from the figure that after improvement, the average precision oscillation of the model is weakened and tends to be stable quickly, and the average precision curve of YOLO-EAF is better than that of YOLOv8n.

### 3.6. Visual Comparison of Detection Effect between YOLO-EAF and YOLOv8n

**Figure 12:**
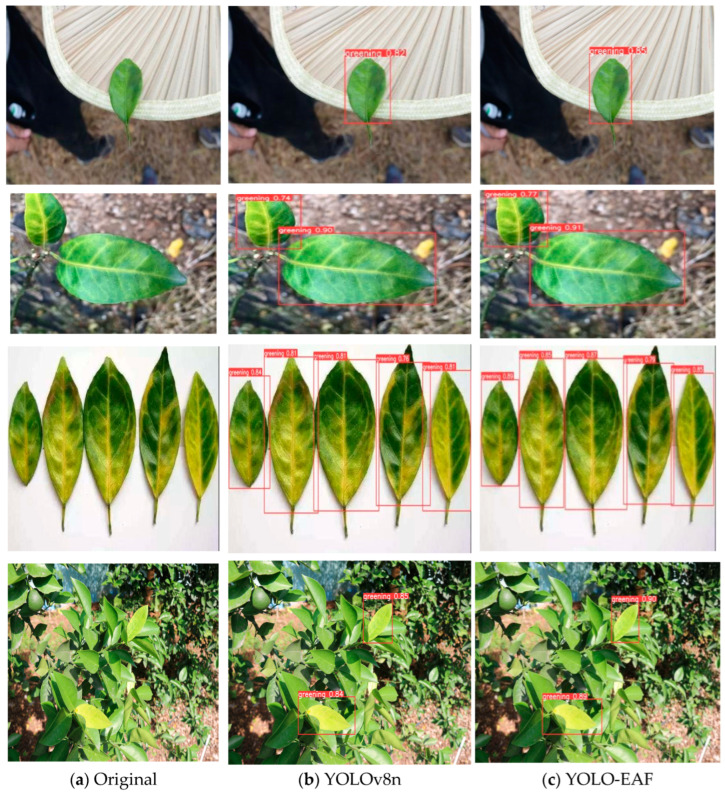
Comparison of YOLO-EAF and YOLOv8n model test results.

In order to qualitatively evaluate the detection effect of the YOLO-EAF model, YOLO-EAF and YOLOv8n were used to visually compare the detection results of citrus Huanglongbing images in the test set. The visualization results are shown in Figure 12. It can be seen from Figure 10 that compared with YOLOv8n, the detection frame of YOLO-EAF contains the whole blade, and the detection results are more accurate than before. At the same time, the confidence of the YOLO-EAF model is higher than that of the YOLOv8n model. Through improvements, the YOLO-EAF model can capture target features in object detection tasks better, enhancing the accuracy and robustness of object detection. It can also better adapt to complex scenes and targets of different sizes, providing higher detection performance.

**Figure 13:**
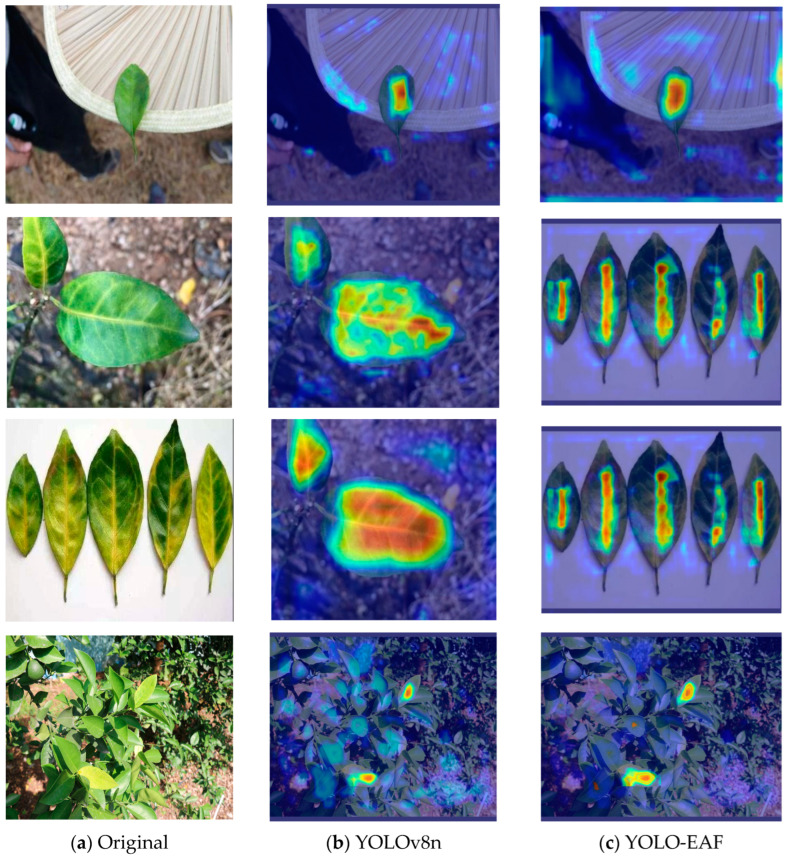
YOLO-EAF and YOLOv8n detection effect heat map comparison.

The visualized heat map can visually display the areas on the image that the model considers to contribute greatly to the object detection task, which can be used to explain the results of the model prediction. In this paper, Grad-CAM [43] was used to generate the object detection heat map. Grad-CAM is a gradient-based neural network visualization tool. We used the gradient information of the last convolutional layer to calculate the weight of each channel and map the weighted feature map to the original image in the form of a heat map. The detection heat map results of some citrus Huanglongbing images are shown in Figure 13. It can be seen from the thermal region shown in the figure that the deeper the red, the greater the weighted feature weight. Also, it can be seen that the thermal value of YOLO-EAF is higher than that of the YOLOv8n model, and the improved model pays more attention to the target area.

### 3.7. Comparison of Different Models

Through a series of improvements to YOLOv8n, we achieved an increase in accuracy. To further validate the recognition performance of YOLO-EAF, we conducted experimental comparisons with other mainstream object detection models on the same dataset. We compared different models in terms of precision (P), recall (R), mean average precision (mAP@0.5), $F_{1}$-score, model parameters, Giga Floating Point Operations, and frames per second on the test set. The results are presented in Table 4.

It can be seen from Table 4 that after the images are augmented, the detection accuracy of different detection methods is improved. Compared to other models, YOLO-EAF achieved superior performance on the test set in terms of precision, mAP(0.5), and F1-score, with values of 82.7%, 84.7%, and 77.83%, respectively. Its mAP(0.5) improved by 29.73, 27.43, 5.7, 4.5, 3.3, 9.6, and 6 percentage points when compared to the SSD, Faster-RCNN, YOLOv5s, YOLOv7-tiny, YOLOv8n, YOLOv9t [44], and YOLOv10n [45] models, respectively. The parameters and computational complexity (GFLOPs) of the YOLO-EAF model are comparable to those of YOLOv8n. Therefore, YOLO-EAF has the best comprehensive performance and can be used to realize the rapid and accurate identification of citrus Huanglongbing in natural environments.

**Figure 14:**
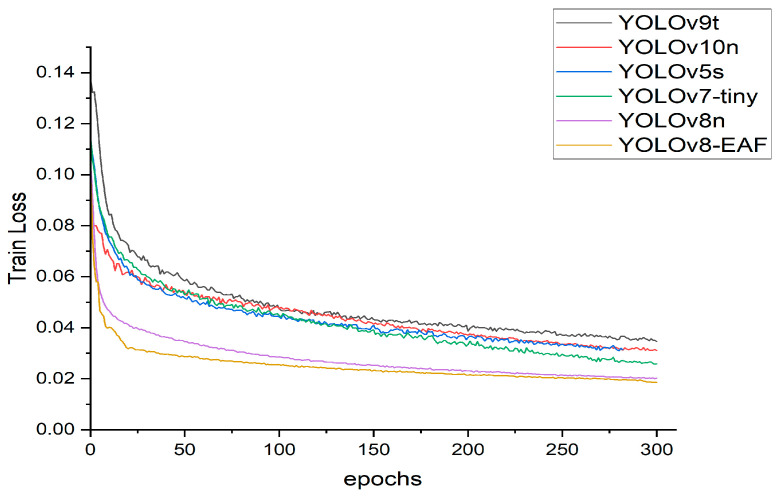
During network training, compare the downward trend of the loss function in the YOLO series in the experiment. Compared with other YOLO models, YOLO-EAF’s loss drops faster.

Figure 14 shows the training loss curve of the YOLO model in the comparative experiment. The change trends of the loss value with the number of iterations reflect the training effect of the model. The closer the loss value is to 0 after the training, the more the model can learn the true distribution of the data. It can be seen intuitively from the curve in the figure that the loss values of each model decrease with the increase in the number of training iterations and gradually tend to be stable. After 150 iterations of YOLO-EAF, the training loss value gradually converges and is less than 0.02. Compared with other models, YOLO-EAF not only converges faster but also has a loss value closer to 0, which indicates that the YOLO-EAF model has a better detection effect and generalization ability.

## 4. Conclusions

In natural environments, traditional object detection networks still struggle to effectively detect Huanglongbing leaves in citrus orchards with scattered distributions of complex background noise, especially those with small targets, occlusions, and high similarity to other citrus diseases. To efficiently detect Huanglongbing, this paper proposes a novel citrus Huanglongbing detection network named YOLO-EAF by improving the structure of the original YOLOv8n. It incorporates an EMA attention mechanism module into the backbone, encoding global information through dimensional interactions and further aggregating pixel-level features. This allows it to accurately distinguish target and non-target information, reducing interference from non-targets. At the same time, an adaptive spatial feature fusion module is introduced into the neck part, which enhances the importance of key levels by assigning different spatial weights to features at different levels during the multi-level feature fusion process, thus reducing the influence of conflicting information between Huanglongbing targets of different scales. Moreover, replacing the loss function with Focal–EIOU can reduce the missing and false detections of Huanglongbing targets in dense scenarios. In the detection of citrus Huanglongbing, YOLO-EAF achieved precision, recall, mAP (0.5), and $F_{1}$-score of 82.7%, 73.5%, 84.7%, and 77.83% respectively. Compared with the current mainstream object detection models SSD, Faster-RCNN, YOLOv5s, YOLOv7-tiny, YOLOv8n, YOLOv9t, and YOLOv10n, YOLO-EAF achieves the best results in terms of precision and mAP (0.5). The visualization results of model detection show that the YOLO-EAF model proposed in this paper can provide a new approach for the precise identification of citrus Huanglongbing and simplify the orchard management process.

Although the method proposed in this paper improves the detection accuracy of citrus Huanglongbing compared to the original YOLOv8n, the detection speed is slightly reduced. Therefore, establishing how to improve detection accuracy while increasing detection speed is an important direction for future research. With the continuous advancement of technology and the in-depth development of smart agriculture, we believe that by adjusting the network structure, introducing new training techniques, and optimizing algorithms, we can further improve the performance of the model, especially its detection capability in complex scenarios. In the future, we will focus on optimizing the detection accuracy and speed of the model. Meanwhile, since the citrus psylla is the transmission vector of Huanglongbing, we will adopt social network theory and technology to study its pest transmission mechanism and develop corresponding prevention and control techniques, aiming to reduce and control the hazards of citrus Huanglongbing.

## Figures and Tables

**Table 1:** Comparison of recognition results of different attention modules.

Attention	P%	R%	mAP%	F1-Score%	Params/M	GFLOPs	FPS
SE	75.7	70.1	75.7	72.79	3	8.1	110.2
CBAM	74.6	72.3	76.9	73.43	3	8.1	103.5
CA	71.2	74.3	78.2	72.72	3	8.1	109.1
EMA	80.7	72.3	82.4	76.27	3	8.1	112.8

**Table 2:** Comparison of recognition results of various loss functions.

Loss Function	P%	R%	mAP%	F1-Score%	Params/M	GFLOPs	FPS
CIOU	74.3	74.7	81.4	74.50	3	8.1	118.4
MPDIOU	75.2	74.4	81.6	74.80	3	8.1	105.3
EIOU	79.2	69.3	81.9	73.92	3	8.1	117.2
Focal–EIOU	79.4	71.9	82.1	75.46	3	8.1	116.7

**Table 3:** Ablation results of the YOLO-EAF network structure on the test set. A check mark (√) indicates whether to enable the specified module.

YOLOv8n	EMA	ASFF	Focal–EIOU	P%	R%	mAP%	F1-Score%	Params/M	GFLOPs	FPS
√				74.3	74.7	81.4	74.50	3	8.1	118.4
√	√			80.7	72.3	82.4	76.27	3	8.1	112.8
√		√		75.4	75.9	83.3	75.65	3	8.2	95.5
√			√	79.4	71.9	82.1	75.46	3	8.1	116.7
√	√	√		75.9	74.8	83.2	75.35	3	8.2	83.3
√	√		√	76.9	75.1	83.7	75.99	3	8.1	109.4
√		√	√	75.3	78.3	84.1	76.77	3	8.2	91.1
√	√	√	√	82.7	73.5	84.7	77.83	3	8.2	94.4

**Table 4:** Comparison results between YOLO-EAF and other mainstream object detection models on the test set.

Model	P%	R%	mAP%	F1-Score%	Params/M	GFLOPs	FPS
SSD	62.39	55.73	54.97	58.87	100.2	125.7	25.2
Faster-RCNN	51.74	64.82	57.27	57.55	108.8	144.2	6.3
YOLOv5s	73.0	75.9	79.0	74.42	7.7	20.9	48.2
YOLOv7-tiny	78.5	73.4	80.2	75.86	6.3	13.2	64.7
YOLOv8n	74.3	74.7	81.4	74.50	3	8.1	118.4
YOLOv9t	74.3	69.5	75.1	71.8	2	7.6	126.1
YOLOv10n	69.7	75.5	78.7	72.5	2.7	8.2	86.8
YOLO-EAF	82.7	73.5	84.7	77.83	3	8.2	94.4

## Data Availability

Data are contained within the article.
